# Biomimetic Mineralization of Tannic Acid-Supplemented HEMA/SBMA Nanocomposite Hydrogels

**DOI:** 10.3390/polym13111697

**Published:** 2021-05-22

**Authors:** Tai-Yu Chen, Shih-Fu Ou, Hsiu-Wen Chien

**Affiliations:** 1 Department of Chemical and Materials Engineering, National Kaohsiung University of Science and Technology, Kaohsiung 807618, Taiwan; terry25197@gmail.com; 2 Department of Mold and Die Engineering, National Kaohsiung University of Science and Technology, Kaohsiung 807618, Taiwan; m9203510@nkust.edu.tw; 3 Photo-Sensitive Material Advanced Research and Technology Center (Photo-SMART Center), National Kaohsiung University of Science and Technology, Kaohsiung 807618, Taiwan

**Keywords:** hydrogels, HEMA, zwitterions, biomimetic mineralization, antibacterial

## Abstract

This study developed a tannic acid (TA)-supplemented 2-hydroxyethyl methacrylate-co-sulfobetaine methacrylate (HEMA-co-SBMA) nanocomposite hydrogel with mineralization and antibacterial functions. Initially, hybrid hydrogels were synthesized by incorporating SBMA into the HEMA network and the influence of SBMA on the chemical structure, water content, mechanical properties, and antibacterial characteristics of the hybrid HEMA/SBMA hydrogels was examined. Then, nanoclay (Laponite XLG) was introduced into the hybrid HEMA/SBMA hydrogels and the effects evaluated of the nanoclay on the chemical structure, water content, and mechanical properties of these supplemented hydrogels. The 50/50 hybrid HEMA/SBMA hydrogel with 30 mg/mL nanoclay showed outstanding mechanical properties (3 MPa) and water content (60%) compared to pure polyHEMA hydrogels. TA then went on to be incorporated into these hybrid nanocomposite hydrogels and its effects investigated on biomimetic mineralization. Scanning electron microscopy (SEM) and energy-dispersive X-ray spectroscopy (EDX) showed that bone-like spheroidal precipitates with a Ca/P ratio of 1.67% were observed after 28 days within these mineralized hydrogels. These mineralized hydrogels demonstrated an almost 1.5-fold increase in compressive moduli compared to the hydrogels without mineralization. These multifunctional hydrogels display good mechanical and biomimetic properties and may have applications in bone regeneration therapies.

## 1. Introduction

Hydrogels are characterized by their three-dimensional networks of various polymers. Most hydrogels are made up of mostly water, which makes them suitable for the development of various biomaterials, especially those used in soft tissue engineering [1]. In the last few years, there has been an increasing interest in the beneficial properties of hydrogels and their application as engineered scaffolds for mineralized tissues [2,3,4,5]. However, their application in hard tissue engineering is less common, as hydrogels are known to exhibit several disadvantages in these settings, including their inherently weaker mechanical properties and poor mineralization upon implantation [4]. In addition, hydrogels are difficult to sterilize due to their high water content [6]. Therefore, the preparation of high-strength hydrogels that possess the capacity to mineralize and have some degree of antibacterial effect have garnered the most interest.

Non-ionic poly(2-hydroxyethyl methacrylate) (polyHEMA) hydrogels have been widely adopted in various biomedical applications because of their good biocompatibility, hydrophilicity, and excellent mechanical properties [7,8]. However, the hydration of these polyHEMA materials tends to be lower than that of native tissues. Their low hydrophilicity limits cellular growth into them. This means that it is fairly common for researchers to incorporate a variety of hydrophilic materials, such as methacrylic acid, *N*-vinyl-2-pyrrolidone, poly(ethylene glycol) methacrylate, or acrylamide into the polyHEMA hydrogel networks to compensate for this issue [9,10,11]. Recently, zwitterionic poly(sulfobetaine methacrylate) (polySBMA), which carries both positively and negatively charged ions in a chemical structure, has attracted interest. The presence of both ions allow these hydrogels to maintain electrical neutrality and produce strong interactions with water molecules, resulting in high hydration and non-fouling properties [12,13,14]. In addition, sulfobetaine ligands can also act as effective mineral nucleating sites [15]. However, the weak mechanical properties of polySBMA hydrogels limit their application [16]. Therefore, the incorporation of SBMA into the HEMA network facilitates the creation of additional crosslinking networks improving overall water content and mechanical properties in these hybrid hydrogels.

To date, many strategies have been reported to reinforce the mechanical performance of conventional hydrogels, including double-network, nanocomposite, macromolecular microsphere composite, and supramolecular hydrogels [17]. This list includes nanoclay nanocomposite hydrogels which can be prepared through in situ free-radical polymerization making the incorporation of nanoclay materials easy, convenient, and low-cost. Laponite XLG nanosilicates (Na_0.7_^+^[(Mg_5.5_Li_0.3_)Si_8_O_20_(OH)_4_]_0.7_^−^) are a synthetic disk-shaped smectite, whose incorporation into polymer matrices not only enhances their mechanical properties but also stimulates osteogenic differentiation [18,19]. This is because the divalent cations such as Mg^2+^ from the Laponite XLG substrates mediate improved cellular adhesion and the activation of complex gene transduction pathways, leading to cell differentiation and osteogenesis [19].

**Figure 1:**
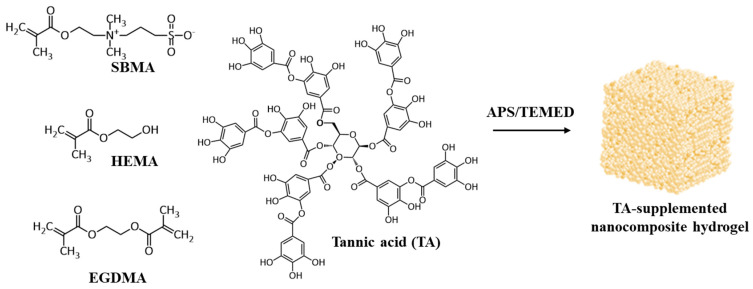
Schematic illustration of the formation of TA-supplemented HEMA/SBMA nanocomposite hydrogel via free radical reaction.

Given the various advantages of the aforementioned materials, it was hypothesized that hybrid nanocomposite hydrogels incorporating HEMA, SBMA and nanoclay should act as suitable scaffolds for bone regeneration. However, a previous study showed that polySBMA hydrogels are effective for mineralization but only under very specific pH and temperature conditions [15]. Therefore, it was tried to incorporate tannic acid (TA) within these nanocomposite hydrogels to promote mineralization under biomimetic conditions. Tannic acid is a member of the phenol family and contains multiple 3,4,5-trihydroxybenzoyl (galloyl) groups arranged in a star-shape [20]. Its chemical formula is often given as C_76_H_52_O_46_, which corresponds to the number of galloyl moieties per molecule and ranges from 2 to 12 depending on the plant source used to extract the tannic acid. Literature indicates that TA can form insoluble metal complexes with various metal ions, such as Fe^3+^ and Al^3+^ [21,22]. A previous study indicated that the catechol group accelerates hydroxyapatite formation by mediating the co-precipitation of calcium and phosphate ions [23]. In addition, TA is a gallol derivative, which is a strong candidate for developing nucleation sites for mineralization. Therefore, this aim of the study tried to design a high strength and antibacterial TA-supplemented nanocomposite hydrogel (Figure 1) and evaluate its application as a scaffold for osteogenesis. First, SBMA was incorporated into the HEMA network and the influence examined of SBMA on the water content, mechanical, and antibacterial properties of the hybrid HEMA/SBMA hydrogels. Then, nanoclay was incorporated into the hybrid HEMA/SBMA hydrogels and the effect evaluated of nanoclay on the reinforcement of the mechanical properties of these materials. Finally, in order to bioactivate the nanocomposite hydrogels, TA went on to be added to the gels and its effects evaluated on mineralization. These novel TA-supplemented HEMA/SBMA nanocomposite hydrogels demonstrated high strength, low fouling, and osteogenic properties for the potential application in the scaffold of bone regeneration.

## 2. Materials and Methods

### 2.1. Materials

2-Hydroxyethyl methacrylate (HEMA, 97%), ethylene glycol dimethacrylate (EGDMA, 98%), ammonium persulfate (APS, 98%), silver nitrate (AgNO_3_, 99.85%), and tris(hydroxymethyl)aminomethane (Tris, 99.8%) were purchased from ACROS (Geel, Belgium). [2-(Methacryloyloxy)ethyl]dimethyl-(3-sulfopropyl)ammonium hydroxide (SBMA, 98%) was purchased from Hopax Fine Chemicals (Kaohsiung, Taiwan). Tannic acid (TA 97%) and *N*,*N’*,*N’*,*N’*-tetramethylethylene-1,2-diamine (TEMED, 99%) were purchased from Alfa Aesar (Ward Hill, MA, USA). Inorganic clay (Laponite XLG) was provided by BYK (Wesel, Germany). *Staphylococcus aureus* (*S. aureus*, ATCC 21351) was obtained from the Food Industry Research and Development Institute (Hsinchu, Taiwan); these bacteria were maintained in Trypticase soy agar containing 15 g/L agar (BD, Franklin Lakes, NJ, USA), 15 g/L tryptone (Cyrusbioscience, Taipei, Taiwan), 5 g/L soy peptone (Cyrusbioscience, Taipei, Taiwan), and 5 g/L NaCl. The bacterial suspension buffer was prepared using 0.85% NaCl at pH 7.

### 2.2. Preparation of Hydrogel Materials

Initial hydrogel polymerization was completed using APS/TEMED which relies on free-radical mediated polymerization at room temperature. The composition of the hydrogels and their relative weight ratios are described in Tables S1–S3 (in supplementary materials). For the preparation of the hybrid HEMA/SBMA hydrogels, HEMA, SBMA, and EGDMA were dissolved in tris buffer solution (pH = 8.5, 10 mM) followed by the addition of 5 mg APS and 5 μL TEMED to initiate polymerization. For the preparation of the hybrid HEMA/SBMA nanocomposite hydrogels, HEMA, SBMA, and EGDMA were dissolved in one-third of tris buffer solution, while Laponite XLG were dissolved in two-thirds of tris buffer solution. After the two solutions were completely dissolved, the two solutions were mixed, then 5 mg APS and 5 μL TEMED were added to initiate polymerization. For the preparation of the TA-supplemented hybrid HEMA/SBMA nanocomposite hydrogels, HEMA, SBMA, EGDMA, and TA were first dissolved in one-third of the tris buffer solution, while Laponite XLG was dissolved in two-thirds of the tris buffer solution. After the two solutions were completely dissolved, the two solutions were mixed evenly and 5 mg APS and 5 μL TEMED added to initiate polymerization. Each reaction solution was poured into two PP plates separated by a 2 mm thick spacer. After 24 h reaction, the copolymers were immersed in DI water several times to allow for hydration and the removal of residual unreacted reagents.

### 2.3. Characterization

The chemical structures of the hydrogels were evaluated by Fourier transform infrared (FTIR) spectroscopy (Spectrum One, PerkinElmer, Waltham, MA, USA) using an attenuated total reflection (ATR) diamond crystal accessory. FTIR spectra were scanned in the absorbance mode from 4000 to 650 cm^−1^ at a resolution of 4 cm^−1^, and 48 cumulative scans were collected.

The fully swollen hydrogels were weighed and dehydrated in an oven at 100 °C overnight. Dried hydrogel disks were weighed and their equilibrium water content (EWC) was calculated using the following equation (*m*_s_ − *m*_d_)/*m*_s_ × 100(%), where *m*_s_ is the mass of the fully swollen hydrogel and *m*_d_ is the mass of the dry hydrogel [24].

For calculating the volume fraction of the swollen hydrogels, the hydrogels after synthesis were first cut specimens into a cylindrical form and the diameter of the hydrogel samples measured by a digital caliper. After that, the hydrogels were immersed in DI water for full swelling and the diameter measured of the swelling hydrogel. The volume fraction was calculated as (*D*/*D*_o_)^3^, where *D* and *D*_o_ are the diameter of hydrogels after equilibrium swelling in water and after synthesis, respectively [16].

A texture analyzer (EZ-SX, Shimadzu, Japan) with a 500 N load cell was used to measure the Young’s modulus of each hydrogel. Hydrated materials with a thickness of 2 mm were cut into 5 mm in diameter sections and placed on the stage at room temperature. The hydrogels were then evaluated at a compressive rate of 1 cm/min.

### 2.4. Evaluation of Bacterial Attachment

The antibacterial properties of the hydrogels were evaluated using the *S. aureus* attachment. First, the hydrogels were used to coat a 96-well tissue culture plate, then 100 μL of 10^6^ CFU/mL *S. aureus* was added to each well and incubated at 37 °C for 1 h. The wells were then rinsed with sterile saline and the surviving bacteria were stained using SYTO 9 (S34854, Invitrogen) to determine bacterial attachment. These experiments were visualized using a fluorescence microscope (Zeiss, Oberkochen, Germany). The photographs were further analyzed using ImageJ software to quantify the fluorescence intensity and evaluate the bacterial attachment in each experiment.

### 2.5. Mineralization

The contact of bone-substituting biomaterials with the biological environment is directly related to the success of the osteointegration process. Among them, in vitro tests using simplified model fluids such as Hanks’ Balanced Salt Solution (HBSS) can be practiced as a first step to evaluate the effects of implantable materials in vivo [25]. The composition of HBSS contained many inorganic ions, such as Ca^2+^ and HPO_4_^2−^, which was assumed to form complexes with TA. Briefly, the TA-supplemented hydrogels were immersed in 2 mL of Hank’s balanced salt solution (1x HBSS, Gibco, Cat No. 14025076) and subject to continuous stirring at 37 °C for 4 weeks [26]. HBSS was replenished every two days and samples were removed at various intervals, rinsed with DI water and dried in an oven at 50 °C, then stored for subsequent analysis. Hydrogel mineralization was observed using a scanning electron microscope (SEM, HITACHI S-3000N, Tokyo, Japan). Elemental analysis was also performed using an SEM equipped with an energy dispersive X-ray spectrometer (EDX, HORIBA EX-250, Tokyo, Japan).

### 2.6. Statistical Analysis

The data are reported as mean ± standard deviation (SD). The statistical analyses of different groups were performed using the Student’s t-test. Probabilities *p* ≤ 0.05 were considered statistically significant. All statistical analyses were performed using the GraphPad Instat 3.0 program (GraphPad Software, San Diego, CA, USA).

## 3. Results

### 3.1. Preparation of Hybrid HEMA/SBMA Hydrogels

**Figure 2:**
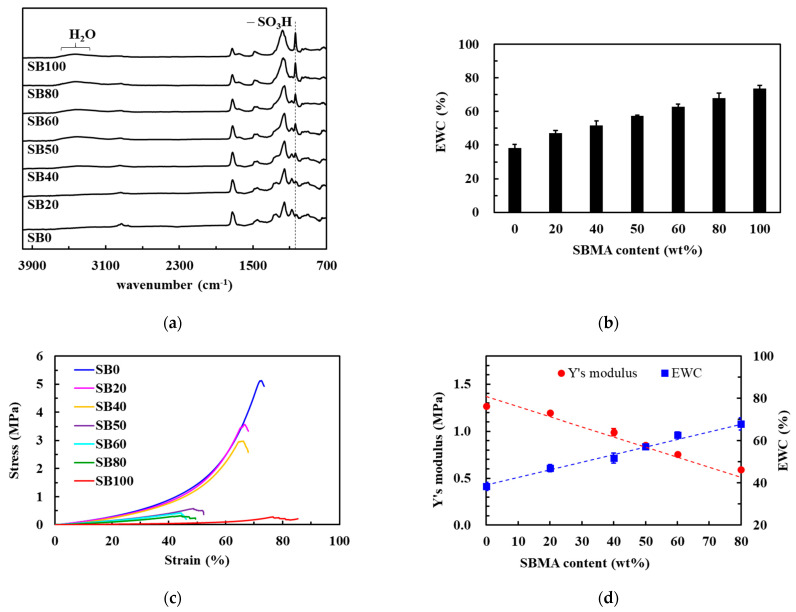
Preparation of hybrid HEMA/SBMA hydrogels. (**a**) FTIR analysis of the hybrid hydrogels. (**b**) The percentage of EWC as a function of the SBMA ratio in the hybrid hydrogels. (**c**) The stress-strain curves for the hybrid hydrogels with various ratios of SBMA. (**d**) Young’s moduli and EWC values for the hybrid hydrogels with various ratios of SBMA. Value = mean ± standard deviation, *n* = 5.

PolyHEMA hydrogels are hydrophilic polymers that have been widely used in many biological and medical applications. However, a low EWC (less than 40%) limits their utility. To improve the performance of polyHEMA hydrogels, the hydrophilic monomer, SBMA, which can bind water molecules via ionic-induced hydration, was added [12]. First, the different ratios of HEMA and SBMA to investigate their properties were evaluated (Table S1). Pure polyHEMA hydrogel (i.e., SB0) was shown to produce several strong, characteristic adsorption bands at 1410–1100 cm^−1^, corresponding to the C-O stretching vibration and O–H in-plane bending vibration (Figure 2a). After incorporating SBMA, the FTIR spectra revealed a new peak at 1038 cm^−1^, corresponding to the –SO_3_H group. It is worth noting that a new broad band from 3600–3000 cm^−1^ was also observed when the SBMA content was increased likely as a result of increased water uptake by the SB moieties from the atmosphere.

**Figure 3:**
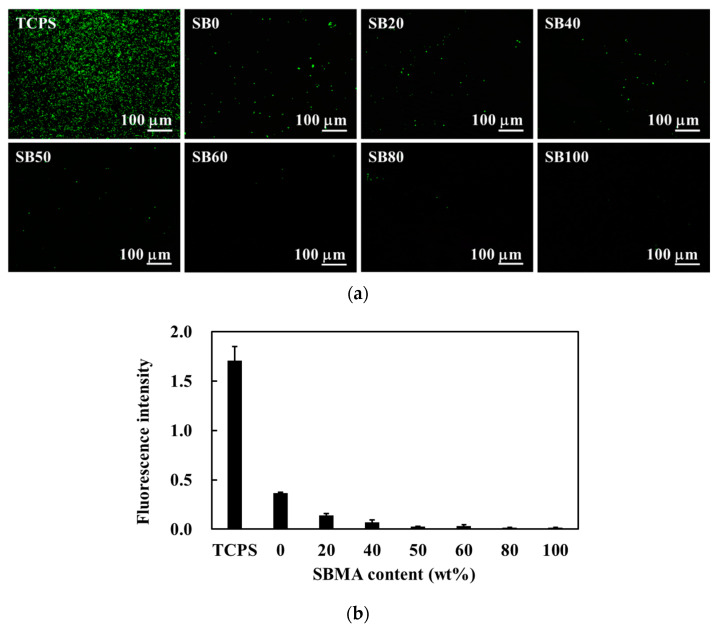
(**a**) Fluorescence images of *S. aureus* attachment to the hybrid HEMA/SBMA hydrogels. (**b**) The fluorescence intensity was analyzed using Image J. Value = mean ± standard deviation, *n* = 5.

The results of the EWC experiments (Figure 2b) confirmed this increased water uptake following the inclusion of more SB groups. Pure polyHEMA hydrogels exhibited a low EWC of only 38% but SBMA incorporation was shown to increase EWC in a dose dependent manner, likely via the ionic interactions between the SB groups and the water molecules in the environment [27]. These highly hydrated polymers also displayed an increase in their non-fouling properties [28]. Pure polyHEMA hydrogels already exhibit resistance to bacterial attachment (Figure 3) but this was further enhanced by the addition of the SBMA polymers. The hydration and high EWC of these hydrogels results in improved antifouling function by preventing initial bacterial attachment, which likely prevents any subsequent biofilm formation. Thus, the bacterial-resistance properties of these hybrid hydrogels mean that they are suitable for tissue engineering.

Although adding SBMA to the hydrogels improves EWC and resistance to bacterial attachment, SBMA incorporation does weaken the mechanical properties of these gels. Pure polyHEMA hydrogel demonstrates a strong compressive modulus (1.26 ± 0.02 MPa), while pure polySBMA hydrogels show a very weak compressive modulus (0.17 ± 0.01 MPa) owing to the highly hydrophilic pendant groups of SBMA (Figure 2c,d). Therefore, as the SBMA concentration increased, so the compressive moduli of the hybrid hydrogels decreased. Given our need to balance the EWC and mechanical properties, the SB50 ratio was chosen to continue our study using.

### 3.2. Preparation of Nanocomposite Hydrogels

**Figure 4:**
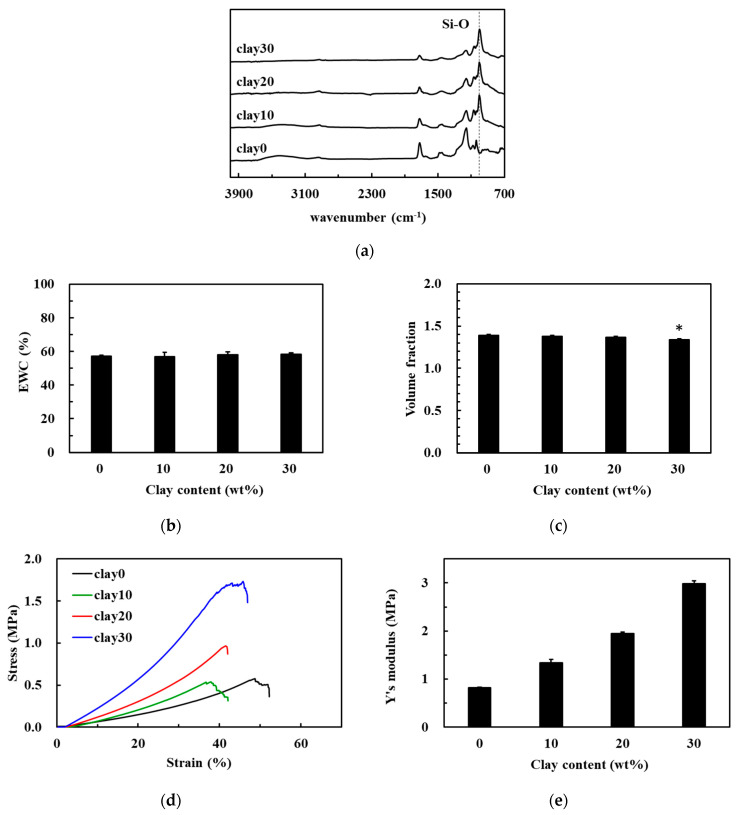
Preparation of hybrid HEMA/SBMA nanocomposite hydrogels. (**a**) FTIR analysis of the nanocomposite hydrogels. The percentage of EWC (**b**) and the volume fraction (**c**) as functions of clay content in the nanocomposite hydrogels. (**d**) The stress-strain curves for the nanocomposite hydrogels produced using various contents of clay. (**e**) Young’s moduli of the nanocomposite hydrogels with various contents of clay. Value = mean ± standard deviation, *n* = 5. *: *p* < 0.05 vs. clay0.

Given this compromise in the mechanical integrity of the hydrogels, the addition of nanoclay as a means of improving the mechanical properties of these hybrid HEMA/SBMA hydrogels went on to be evaluated. The nanoclay was dispersed within the SB50 gels at concentrations of 0, 10, 20, and 30 mg/mL, (clay0, clay10, clay20, and clay30) and then evaluated (Table S2). The FTIR spectrum confirmed the successful synthesis of the nanocomposite hydrogels due to the appearance of a new peak at 993 cm^−1^ (Figure 4a), which corresponds to the Si–O stretching band [29]. The EWC always maintained in the range of about 58% after introducing nanoclay in the polymeric matrix (Figure 4b); however, it is noted that a cognate decrease in the volume fraction when very high concentrations of the nanoclay were included in the polymer matrices (Figure 4c). These results were similar to those of a previous study that evaluated a polyethylene glycol/Laponite hydrogel system [29]. This could be due to an increase in the overall crosslinking density in these systems resulting from novel interactions between the polymer chains and the nanoclays [29].

The compressive stress value for the clay0 hydrogels was 0.32 MPa at 35% compressive strain (Figure 4d). However, increasing nanoclay content increased the resistance of these hydrogels to compressive stress with caly10, clay20, and clay30 polymers achieving compressive strain values of 0.49, 0.71, and 1.33 MPa at 35% strain, respectively. In addition, the compressive modulus of the clay0 hydrogels was 0.82 ± 0.01 MPa (Figure 4e). However, when the clay content was increased to 10 or 20 mg/mL, it is noted that a concurrent increase in the compressive modulus of these nanocomposite hydrogels, 1.34 ± 0.07 MPa and 1.95 ± 0.03 MPa, respectively. The compressive modulus of clay30 was 2.99 ± 0.06 MPa, indicating that the compression modulus was 3.65 times higher than that of clay0 hydrogels. This can be attributed to the production of complex chemically crosslinked networks and the secondary interactions between the polymer and nanoclay [30,31].

### 3.3. Preparation of TA-Supplemented Nanocomposite Hydrogels for Mineralization

This means that clay30 may be the ideal hydrogel matrix for bone transplants as it gives a good balance to the mechanical and hydration properties needed to facilitate proper regenerative repair. However, these hydrogels lack nuclear sites for mineralization under biomimetic conditions, preventing the creation of chemical bonds with the bone. To remedy this, the nanocomposite hydrogels with TA went on to be supplemented. Previous studies have suggested that catechol groups can generate radicals in an oxidative state, which can act as initiators for the free radical polymerization of (methyl) acrylic monomers [32,33]. Under alkaline conditions, dopamine is first oxidized to dopaminoquinone and then transferred to semi-quinone radical species via a single-electron exchange reaction. These radicals act as reactive species initiating the free-radical polymerization of acrylate monomers to form polymers (Figure S1 in supplementary materials) [32]. Therefore, it was hypothesized that the gallol groups in TA could generate reactive radicals in tris-base solutions initiating the crosslinking of HEMA, SBMA, and EGDMA through a radical chain propagation mechanism, and bond to the polymer network of hydrogels.

**Figure 5:**
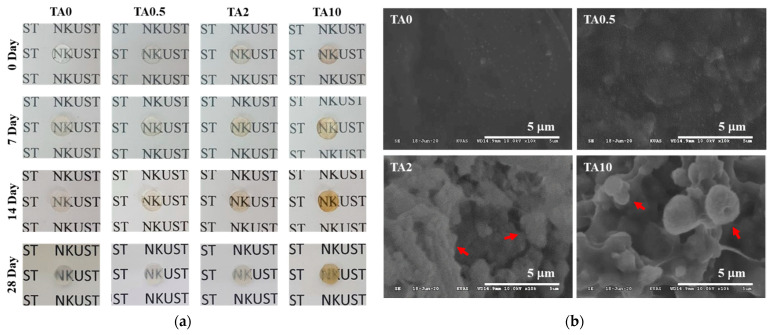
(**a**) Photographs of the TA-supplemented nanocomposite hydrogels before and after mineralization in HBSS. (**b**) SEM images of the surface of the hydrogels following 28 days of mineralization. The bone-like spheroidal precipitates were observed in TA2 and TA10 hydrogels (red arrows). The scale bar represents 5 μm.

It was also hypothesized that the gallol groups could interact with the calcium ions in the bone minerals using coordination bonding. Therefore, the effect of adding varying concentrations of TA on the mineralization of these nanocomposite hydrogels was investigated. Four different TA concentrations were evaluated and the TA-supplemented nanocomposite hydrogels were soaked in HBSS for 7, 14, and 28 days (Table S3). All TA-supplemented nanocomposite hydrogels were transparent before mineralization (Figure 5a) were transparent, but the opacity of the hydrogels increased as the TA concentration and HBSS immersion (7, 14, and 28 days) increased. This was most obvious in the 28 days samples and it is noted that while TA0 and TA0.5 remained translucent, the TA2 and TA10 hydrogels became opaque at this time point, indicating a significant degree of mineralization in these samples.

SEM images revealed that the surface of the TA0 hydrogel remained smooth and incorporated only a small amount of precipitate (Figure 5b). It is worth noting that the typical bone-like spheroidal precipitates of calcium phosphate (the precursor phase of hydroxyapatite) were homogeneously dispersed across the surface of TA2 and TA10 after immersion in HBSS solution for 28 days (Figure 5b) [23,34]. It was speculated that the gallol moieties form a complex with Ca^2+^ when the TA in the hydrogels are oxidized and that this is the underlying mechanism supporting the nucleation and crystallization of calcium phosphate in these samples. These sites then interact with PO_4_^3−^ to enhance this nucleation, resulting in the production of calcium phosphate [23]. This means that more calcium phosphate precipitates produced as increasing the number of gallol active sites (i.e., high TA concentrations). Notably, EDS data showed that the Ca/P ratios of the precipitates in TA2 and TA10 after 28 days were 1.67 and 1.52, respectively, approaching the theoretical ratio of hydroxyapatite (Ca_10_(PO_4_)_6_(OH)_2_, Ca/P = 1.67) [34], while the Ca and P composition of TA0 remained very low (Table 1), indicating that TA2 and TA10 are good bioactive scaffolds for mineralization.

**Figure 6:**
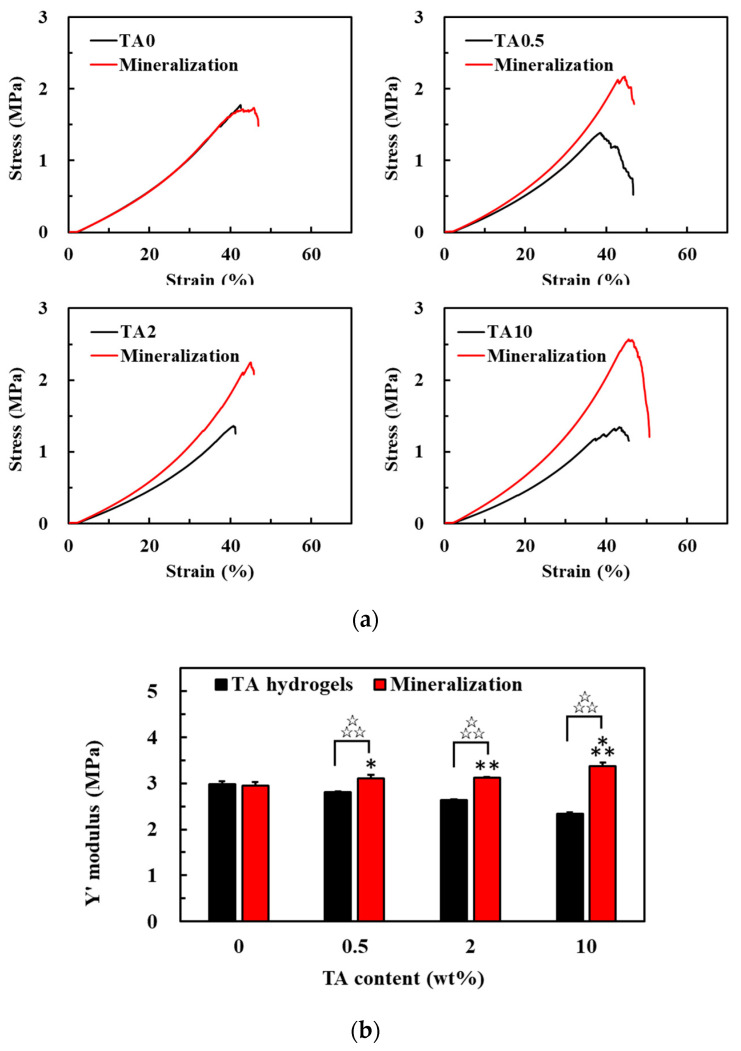
Mechanical properties of the TA-supplemented nanocomposite hydrogels. (**a**) The stress–strain curves for the TA-supplemented nanocomposite hydrogels before and after mineralization. (**b**) Young’s moduli of the TA-supplemented nanocomposite hydrogels before and after mineralization. Value = mean ± standard deviation, n = 4. *: *p* < 0.05 vs. TA0 (mineralization); **: *p* < 0.01 vs. TA0 (mineralization); ***: *p* < 0.001 vs. TA0 (mineralization); ☆☆☆: *p* < 0.001.

On the other hand, the mechanical properties of the TA-supplemented nanocomposite hydrogels before and after mineralization were significantly different. After 28 days of mineralization, the breaking point for the TA0 hydrogels under compressive stress did not obviously change. However, the compressive stress of TA0.5, TA2, and TA10 hydrogels at their breaking points and the compressive strain for each of these hydrogels were increased in comparison to their pre-mineralization values (Figure 6a). The compressive moduli for the original hydrogels were 2.99 ± 0.06, 2.81 ± 0.02, 2.64 ± 0.02, and 2.34 ± 0.02, and after 28 days of mineralization, their compressive moduli were 1-fold-, 1.10-fold-, 1.18-fold-, and 1.44-fold of the original TA0, TA0.5, TA2, and TA10 values, respectively (Figure 6b). This suggests that those hydrogels which were enriched for calcium reinforced their mechanical properties. This is particularly obvious when our results are compared with those from Wu et al., who used gelatin-based hydrogels [35]. They observed that an increased cycle number or longer incubation time in the mineralization solutions resulted in increased compressive moduli for their composite hydrogels [35]. Other reports have suggested that the phenol-derived groups in the polymer network could be used as mineralization scaffolds, wherein in situ mineral nucleation is targeted to the network crosslink sites by strong coordination bonding, thereby preventing the damage to the network elasticity normally observed upon particle incorporation [36]. Taken together this data supports our finding of increased mechanical resilience in response to increased mineralization in our nanocomposite hydrogels.

There have been several attempts at designing a production strategy that resolves the issues around hydrogel mineralization, mechanical strength and hydrophobicity with many successful materials being produced, but all require some compromise. One strategy includes the incorporation of inorganic phases, such as calcium phosphate ceramics and bioglass, into hydrogel matrices [37,38]. These inorganic particles act as nucleation sites that enable further mineralization, thus improving the mechanical properties of the composite material [37,38]. However, the incorporation of these inorganic particles did not have any bonding with the polymer network, which compromises the network elasticity [36]. Another strategy relies on the use of the physiological mineralization process; an example of this is the soaking of hydrogels in saturated calcium phosphate solutions [39,40], or incorporating enzymes in the hydrogels to catalyze the deposition of bone minerals [41,42]. In some cases, this might include the addition of cells or growth factors that facilitate bone morphogenesis into the hydrogel composites to enhance their osteoconductive effects [13]. However, enzymes and growth factors are expensive components, making this a less viable alternative from a commercial perspective. Here, TA was simply incorporated into high-strength HEMA/SBMA nanocomposite hydrogels, which were then able to undergo mineralization and functionalization to improve antibacterial effects. These multifunctional hydrogels have broad application potential in bone tissue engineering and demonstrate that it is possible to produce complex multifunctional hydrogels using readily available, economically viable materials.

## 4. Conclusions

In summary, novel TA-supplemented HEMA/SBMA nanocomposite hydrogels that can facilitate biomimetic mineralization were developed. In this study, the hydration of pure polyHEMA hydrogels and the mechanical properties of chemically crosslinked polySBMA hydrogels were improved by copolymerizing HEMA and SBMA with nanoclay. When these nanocomposites were compared to pure polyHEMA hydrogels (<40% of water content and 1.2 MPa of Young’s modulus), it could be noted that both hydration and mechanical modulus have been significantly improved, bringing them closer to the natural tissue (~60% of water content and 3 MPa of Young’s modulus were recorded in clay30 hydrogels), making these hydrogels more suitable for practical biological applications such as tissue engineering scaffolds. Furthermore, when the TA-supplemented HEMA/SBMA nanocomposite hydrogels were immersed in HBSS, the TA functional group allowed for hydroxyapatite mineralization, which increased mechanical reinforcement. In addition, these biomineralized and functionally enhanced hydrogels retain their mechanical characteristics, making them ideal materials for applications in bone tissue engineering.

## Figures and Tables

**Table 1:** EDX analysis of the hydrogels after 28 days of mineralization.

	Elemental Composition %						
	C	O	Na	Cl	Ca	P	Ca/P
TA0	54.77	42.28	1.90	0.70	0.35	0	-
TA0.5	58.32	38.16	2.98	0	0.22	0.31	0.71
TA2	40.64	48.56	2.50	0.8	4.69	2.81	1.67
TA10	40.62	41.4	2.03	1.14	8.93	5.88	1.52

## Data Availability

Not applicable.

## Supplementary Materials

The following are available online at https://www.mdpi.com/article/10.3390/polym13111697/s1, Table S1: Copolymerization formulations for the hybrid hydrogels with different contents of monomer, Table S2: Copolymerization formulations for the hybrid nanocomposite hydrogels with different contents of clay, Table S3: Copolymerization formulations for the TA-incorporated hybrid hydrogels with different contents of TA, Figure S1: Suggested mechanism of the catechol-triggered polymerization of acrylate monomers.
