# Supplementary material for: Biomimetic Mineralization of Tannic Acid-Supplemented HEMA/SBMA Nanocomposite Hydrogels
Source: Polymers (Basel). 2021 May 22;13(11):1697. doi: 10.3390/polym13111697 (PMC8197008; doi:10.3390/polym13111697)

Table S1 Copolymerization formulations for the hybrid HEMA/SBMA hydrogels with different contents of monomer. For the preparation of the hybrid HEMA/SBMA hydrogels, HEMA, SBMA and EGDMA were dissolved in tris buffer solution (pH = 8.5, 10 mM) followed by addition of 5 mg APS and 5 μL TEMED to initiate polymerization. Each reaction solution was poured into two PP plates separated by a 2 mm thick spacer. After 24 h reaction, the copolymers were immersed in DI water several times to allow for hydration and the removal of residual unreacted reagents.

Name	HEMA (μL)	SBMA (mg)	EGDMA (μL)	Tris (μL)
SB0	600	0	30	400
SB20	480	120	30	400
SB40	360	240	30	400
SB50	300	300	30	400
SB60	240	360	30	400
SB80	120	480	30	400
SB100	0	600	30	400

Table S2 Copolymerization formulations for the hybrid HEMA/SBMA nanocomposite hydrogels with different contents of clay. For the preparation of the hybrid HEMA/SBMA nanocomposite hydrogels, HEMA, SBMA and EGDMA were dissolved in one-third of tris buffer solution (pH = 8.5, 10 mM), while Laponite XLG were dissolved in two-thirds of tris buffer solution. After the two solutions were completely dissolved, the two solutions were mixed, and then 5 mg APS and 5 μL TEMED were added to initiate polymerization. Each reaction solution was poured into two PP plates separated by a 2 mm thick spacer. After 24 h reaction, the copolymers were immersed in DI water several times to allow for hydration and the removal of residual unreacted reagents.

Name	HEMA (μL)	SBMA (mg)	EGDMA (μL)	Clay (mg)	Tris (μL)
Clay0	300	300	30	0	400
Clay10	300	300	30	10	400
Clay20	300	300	30	20	400
Clay30	300	300	30	30	400

Table S3 Copolymerization formulations for the TA-supplemented hybrid HEMA/SBMA nanocomposite hydrogels with different contents of TA. For the preparation of the TA-supplemented hybrid HEMA/SBMA nanocomposite hydrogels, HEMA, SBMA, EGDMA and TA were first dissolved in one-third of tris buffer solution, while Laponite XLG was dissolved in two-thirds of tris buffer solution. After the two solutions were completely dissolved, the two solutions were mixed evenly and added 5 mg APS and 5 μL TEMED to initiate polymerization. Each reaction solution was poured into two PP plates separated by a 2 mm thick spacer. After 24 h reaction, the copolymers were immersed in DI water several times to allow for hydration and the removal of residual unreacted reagents.

Name	HEMA (μL)	SBMA (mg)	EGDMA (μL)	Clay (mg)	TA (mg)	H_2O (μL)
TA0	300	300	30	30	0	400
TA0.5	300	300	30	30	0.5	400
TA2	300	300	30	30	2	400
TA10	300	300	30	30	10	400

Figure S1 Suggested mechanism of the catechol-triggered polymerization of acrylate monomers

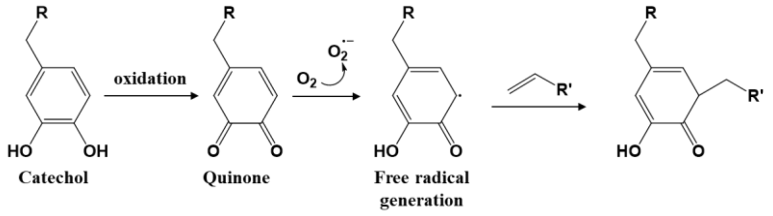

Supplement: Supplementary file 1 [file polymers-13-01697-s001.zip › polymers-1233660-supplementary.pdf]
